# Procalcitonin and Diabetic Foot Ulcer Infections: A Meta‐Analysis

**DOI:** 10.1002/edm2.70066

**Published:** 2025-07-13

**Authors:** Wenqiang Wang, Peilin Zhou, Xinyu Nie, Qikai Hua

**Affiliations:** ^1^ Department of Bone and Joint Surgery (Guangxi Diabetic Foot Salvage Engineering Research Center/Research Centre for Regenerative Medicine) The First Affiliated Hospital of Guangxi Medical University Nanning China; ^2^ Department of Orthopedics The First Affiliated Hospital of USTC, Division of Life Sciences and Medicine, University of Science and Technology of China Hefei China

**Keywords:** diabetic foot ulcers, infection, inflammatory biomarkers, PRISMA guidelines, procalcitonin

## Abstract

**Background:**

Procalcitonin (PCT) is an effective inflammatory marker for diagnosing infection. We assessed the clinical utility of procalcitonin in diagnosing diabetic foot infections.

**Method:**

This meta‐analysis adhered to the PRISMA guidelines. We searched PubMed, Web of Science, Embase and the Cochrane Library for studies on PCT for the diagnosis of diabetic foot published before 1 July 2024. The primary outcome was the standardised mean difference (SMD) in PCT levels between IDFU and non‐IDFU groups, with corresponding 95% confidence intervals (CI). The included studies were cross‐sectional and cohort studies, so the quality of the literature was assessed using the Newcastle–Ottawa Scale (NOS) evaluation criteria. This study's statistical analyses were conducted solely with STATA 15.0 software.

**Result:**

Ten studies comprising 928 patients were ultimately included. There were six cross‐sectional studies and four cohort studies. In total, 532 patients were assigned to the IDFU group and 396 to the non‐infected diabetic foot ulcers (NIDFU) group. The relationship between PCT and DFU was evaluated in ten studies, with significant heterogeneity among the included studies (*x* ^2^ = 54.10, *p =* 0.00001; *I* ^2^ = 83.6%). Therefore, a random effects model was used with a pooled standardised mean difference of 0.79 (95% confidence interval [CI]: 0.43–1.14). The Egger experiment results (*t* = 0.43, *p* = 0.680) indicated that there was no publication bias. Analysis of sensitivity revealed that the results were reliable. Subgroup analyses identified the area as a significant source of heterogeneity. The random‐effects model's meta‐regression results revealed that BMI (*p* = 0.026) and HbA1c (*p* = 0.016) had a significant impact on the heterogeneity of the association between IDFU and PCT levels.

**Conclusion:**

Our study showed a significant correlation between serum PCT levels and IDFU. Identification and treatment of IDFUs as soon as possible can help reduce amputation and mortality rates.

This systematic review and meta‐analysis evaluated the association between serum procalcitonin levels and diabetic foot infections. Ten studies were included, and a random‐effects model showed significantly higher procalcitonin levels in infected patients, supporting its role as a potential diagnostic biomarker for early infection detection in diabetic foot ulcers.

## Graphical abstract

This meta‐analysis of 10 studies involving 928 patients showed that procalcitonin (PCT) levels were significantly higher in patients with infected diabetic foot ulcers (IDFU) compared to those with non‐infected ulcers (NIDFU). Subgroup analysis indicated a stronger association in Asian populations (SMD = 1.15), and meta‐regression identified BMI and HbA1c as major sources of heterogeneity. These findings suggest that PCT may serve as a promising serum biomarker for early identification of infection in diabetic foot ulcers, potentially aiding timely clinical intervention and reducing the risk of amputation and mortality

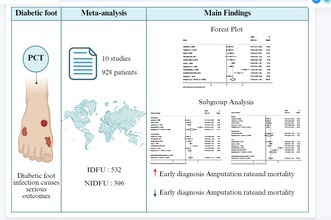

## 1 Introduction

Prolonged hyperglycaemia, leading to vascular and neuropathic complications, is a common issue in diabetes mellitus (DM), often resulting in diabetic foot ulcers (DFU) under conditions like inadequate glycaemic control, uneven pressure distribution or foot deformities [1, 2]. DFU have become a leading cause of clinical amputations, with annual incidence rates ranging from 1.9% to 4.0% and cumulative incidence between 19% and 34% [3]. This condition accounts for one‐third of the annual medical costs associated with DM in the United States, imposing a substantial burden on families and society [4].

Biological markers, particularly inflammatory biomarkers, are vital for assessing normal and pathological processes or responses to therapeutic interventions [5]. The 2019 guidelines for the management of diabetes recommend the early identification of pathogenic microorganisms for infections to determine the classification of infections and mitigate the severe consequences of IDFU [6]. Because of the possibility of progression, necrosis and amputation, prompt diagnosis and treatment of IDFU are essential. Nevertheless, the effects of diabetes on the neurovascular and immune systems may compromise the local response to infection, so clinical symptoms may not be readily apparent [7].

In recent years, numerous new biological inflammatory markers have emerged, including procalcitonin (PCT), C‐reactive protein (CRP), interleukin (IL), pentraxin‐3 (PTX‐3), tumour necrosis factor‐α (TNF‐α) and others [7]. Recent research indicates that serum PCT is becoming a useful biomarker for the diagnosis of IDFU [8]. PCT is a peptide hormone secreted by non‐neuroendocrine parenchymal cells throughout the body, with serum levels below 0.05 ng/mL in healthy individuals and between 50 and 800 ng/mL in patients with severe sepsis. In a study conducted by Asirvatham, serum PCT levels were found to correlate with the presence or absence of infection in DF with greater accuracy than WBC and serum CRP [9].

This meta‐analysis aimed to evaluate the association between serum PCT levels and IDFU diagnosis. By understanding the variation in inflammatory marker levels, early interventions can be implemented to reduce the risk of complications, amputation, and mortality in IDFU patients. Previous studies have confirmed the link between PCT and IDFU, but no comprehensive evaluation of serum PCT levels in IDFU patients has been conducted. Therefore, this meta‐analysis seeked to determine the significance of the relationship between serum PCT levels and IDFU.

## 2 Methods

This meta‐analysis was reported according to the Preferred Reporting Items for Systematic Reviews and Meta‐analyses (PRISMA) guidelines.

### 2.1 Inclusion and Exclusion Criteria

Inclusion criteria: (1) cohort or cross‐sectional study design; (2) studies investigating the relationship between infected diabetic foot ulcers (IDFU) and inflammatory biomarkers; (3) availability of comprehensive and calculable data.

Exclusion criteria: (1) studies with evident bias; (2) findings that are irrelevant or incomplete; (3) studies lacking full‐text availability.

### 2.2 Search Strategy

A comprehensive literature search was conducted in PubMed, Embase, Cochrane Library and Web of Science for studies published before 1 July 2024. The search terms used included: (“Diabetic foot” OR “Foot, Diabetic” OR “Diabetic Feet” OR “Feet, Diabetic” OR “Foot Ulcer, Diabetic”) AND (“infections” OR “Infestation and Infection” OR “Infections and Infestations” OR “Infestation and Infection” OR “Infection”) AND (“Procalcitonin” OR “PCT” OR “Calcitonin Precursor Polyprotein” OR “Calcitonin Related Polypeptide Alpha” OR “Pro‐Calcitonin” OR “Calcitonin‐1” OR “Calcitonin 1”). After the initial search, titles and abstracts were screened for relevance, followed by a full‐text review of studies that met the inclusion criteria to determine the final selection. Two authors independently assessed the relevance of titles, abstracts, and full texts of potentially eligible studies. In cases of disagreement, a third author was consulted to resolve the differences.

### 2.3 Data Collection

Two authors, utilising a standardised protocol for data capture, extracted relevant IDFU‐related information from the final set of included literature. Authors, publication year, country, study design, disease duration, BMI, Hba1c and PCT levels for IDFU and NIDFU were ultimately extracted. The primary outcome variable was the standardised mean difference (SMD) of PCT levels between the IDFU and NIDFU groups, with corresponding 95% confidence intervals (CI).

### 2.4 Quality Assessment

The quality of the included cohort and cross‐sectional studies was assessed using the Newcastle–Ottawa Scale (NOS), a tool with demonstrated high reliability [10]. The NOS evaluates studies based on three domains: selection of study participants (up to four points), comparability of study groups (up to two points) and assessment of exposure or outcome (up to three points), with a maximum score of nine indicating higher study quality and lower risk of bias.

Two reviewers independently applied the NOS to all included studies, and any discrepancies were resolved through discussion until consensus was achieved.

### 2.5 Statistical Analysis

We first generated a forest plot to visualise the relationship between PCT and IDFU. Forest plots assessed heterogeneity, and when *I* ^2^ > 50%, it indicated that the heterogeneity of the included literature was substantial and a random effects model was used; otherwise, a fixed effects model was used for statistical analyses. Publication bias was evaluated using funnel plots and Egger's test. Additionally, sensitivity analyses were performed by sequentially excluding each study to assess its impact on the overall results. Subgroup analysis was conducted, and meta‐regression was performed to investigate the relationship between study characteristics and study outcomes to identify sources of heterogeneity in the included literature. In our investigation, a two‐sided *p* < 0.05 was regarded as statistically significant. Stata 15.0 was utilised to analyse the collected data in this study.

**TABLE 1:** Baseline characteristics of included studies.

Author, year	Country	Study design	Sample size (M/F)	Age (Year)	BMI (kg/m^2^)	Duration of disease (years)	HbA1c (%)
Dhamodharan, U., 2018 [11]	India	Cohort study	110 (65/45)	59.32 ± 8.55	26.70 ± 4.54	NR	9.7 ± 2.56
Jeandrot, A., 2008 [12]	France	Cohort study	93 (56/37)	68.70 ± 39.16	NR	19.88 ± 21.83	7.22 ± 3.31
Van Asten, S. A., 2017 [13]	America	Cross‐sectional	35 (27/8)	50.76 ± 10.58	29.70 ± 5.22	NR	10.08 ± 2.28
Omar, J., 2023 [14]	Malaysia	Cross‐sectional	214 (111/103)	58.01 ± 10.64	NR	NR	8.65 ± 3.81
Jonaidi Jafari, N., 2014 [15]	Iran	Cross‐sectional	60 (31/29)	58.15 ± 10.11	NR	14.5 ± 7.63	7.65 ± 2.24
Todorova, A. S., 2021 [16]	Sofia	Cross‐sectional	76 (57/19)	60.04 ± 10.48	30.08 ± 6.12	16.20 ± 9.47	9.30 ± 2.18
Aslan, S., 2020 [17]	Turkey	Cohort study	81 (46/35)	60.08 ± 11.32	NR	NR	8.93 ± 2.17
Zakariah, N. A., 2020 [18]	Malaysia	Cohort study	128 (82/46)	61 ± 9.72	NR	NR	8.06 ± 2.84
Al‐Shammaree, S. A. W., 2017 [19]	Iraq	Cross‐sectional	55 (37/18)	52.92 ± 6.73	30.53 ± 8.46	11.49 ± 5.39	9.89 ± 1.33
Korkmaz, P., 2018 [20]	Turkey	Cross‐sectional	76 (51/25)	62.61 ± 11.13	NR	13.79 ± 7.93	9.72 ± 2.25

## 3 Results

### 3.1 Study Selection

**FIGURE 1:**
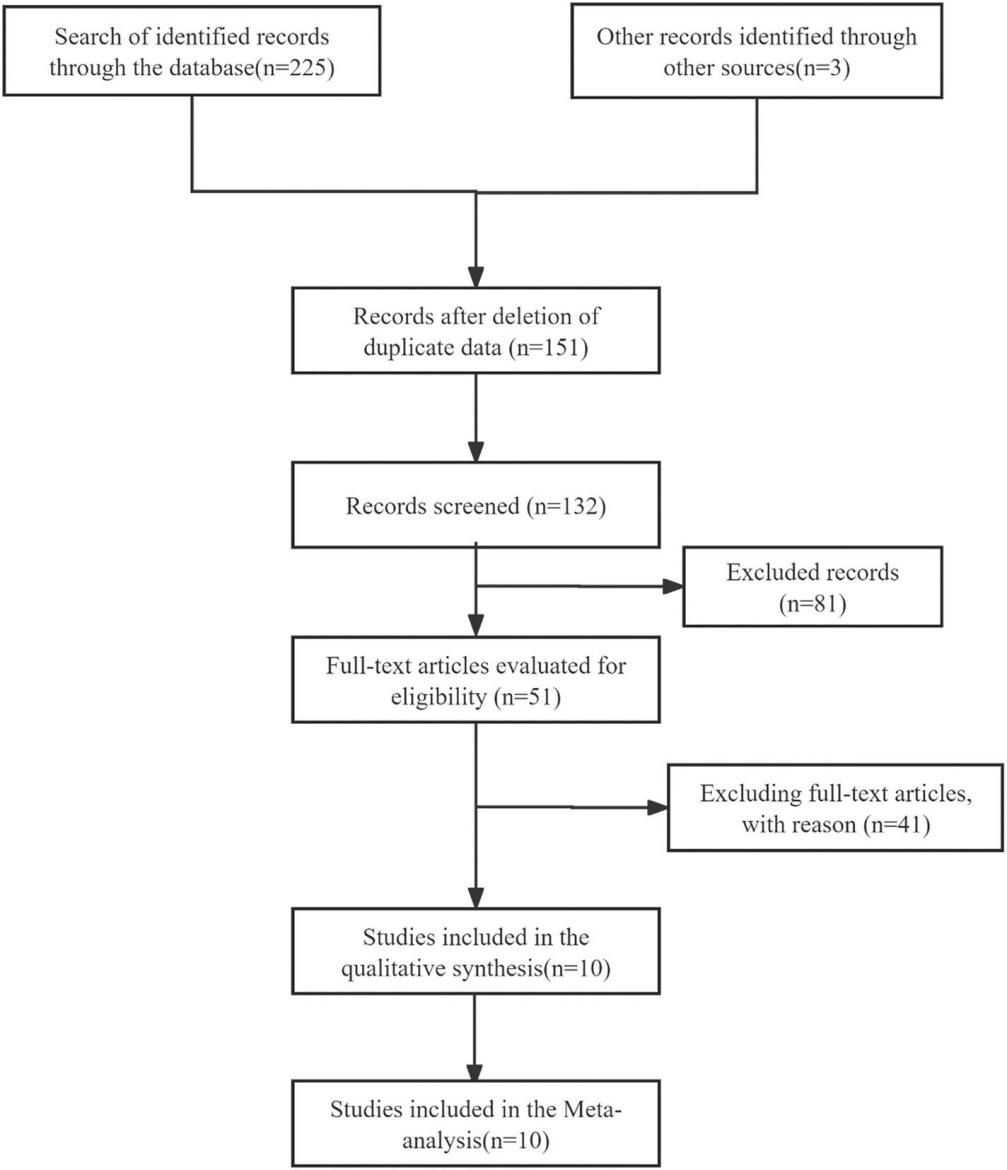
Flow diagram of the article selection process.

Figure 1 depicts the search procedure's outcomes. The initial search strategy yielded 225 results across four databases, and three additional studies were retrieved using alternative methods. After Endnote software had eliminated 77 duplicate articles, 151 titles and abstracts remained. Following the reading of titles and abstracts, 51 complete papers were evaluated based on inclusion and exclusion criteria. Ten articles were chosen for the concluding meta‐analysis.

### 3.2 Study Characteristics

Tables 1 and 2 summarise the characteristics of the ten included studies. These studies recruited a total of 928 patients from eight countries. The mean age was 59.76 years, with 563 male patients. There were 532 patients in the IDFU group and 396 patients in the NIDFU group. Six studies were cross‐sectional and four were cohort studies.

**TABLE 2:** Characteristics of included studies (expanded details).

Author, year	IDFU		NIDFU	
	Mean ± SD	*N*	Mean ± SD	*N*
Dhamodharan., U., 2018 [11]	0.675 ± 0.312	76	0.063 ± 0.003	34
Jeandrot, A., 2008 [12]	0.970 ± 2.771	70	0.085 ± 0.051	23
Van Asten, S. A., 2017 [13]	0.260 ± 0.450	24	0.070 ± 0.070	11
Omar, J., 2023 [14]	0.355 ± 0.467	107	0.077 ± 0.111	107
Jonaidi Jafari, N., 2014 [15]	1.20 0 ± 2.100	30	0.330 ± 0.370	30
Todorova, A. S., 2021 [16]	0.050 ± 0.31	41	0.040 ± 0.023	35
Aslan, S., 2020 [17]	0.630 ± 1.300	48	0.110 ± 0.100	33
Zakariah, N. A., 2020 [18]	0.270 ± 0.510	73	0.050 ± 0.044	55
Al‐Shammaree, S. A. W., 2017 [19]	1.600 ± 1.120	25	0.450 ± 0.090	30
Korkmaz, P., 2018 [20]	0.600 ± 1.700	38	0.150 ± 0.090	38

### 3.3 Quality Assessment

The NOS scores of the literature included in this study are shown in Table 3. The results indicate that the included studies performed well overall, with each of the ten studies presenting minimal risk. Overall, the risk of bias in the included studies was moderate.

**TABLE 3:** NOS quality assessment of included studies.

Study	Selection (stars awarded)	Comparability (stars awarded)	Outcome ascertainment (stars awarded)	Bias risk (total stars awarded)
Dhamodharan, U., 2008 [11]	3	1	3	7
Jeandrot, A., 2008 [12]	3	2	3	8
Van Asten, S. A., 2017 [13]	3	1	3	7
Korkmaz, P., 2018 [20]	3	2	3	8
Jonaidi Jafari, N., 2014 [15]	3	0	3	6
Todorova, A. S., 2021 [16]	3	2	3	8
Omar, J., 2023 [14]	3	1	3	7
Zakariah, N. A., 2020 [18]	3	2	3	8
Al‐Shammaree, S.A.W., 2017 [19]	3	1	3	7
Aslan, S., 2020 [17]	3	2	3	8

### 3.4 Results of Meta‐Analysis

**FIGURE 2:**
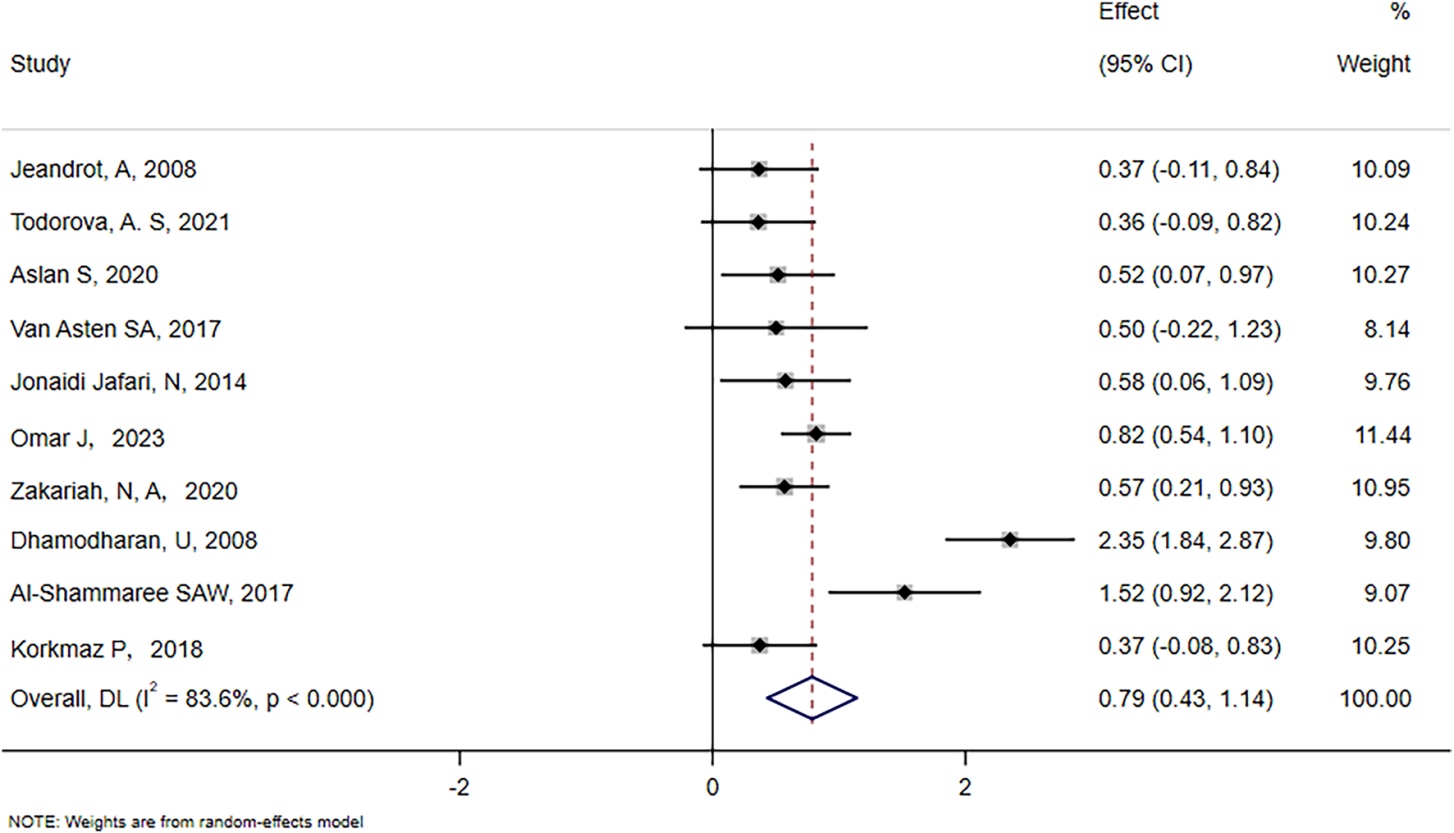
Forest map of PCT levels for IDFU and NIDFU groups.

**FIGURE 3:**
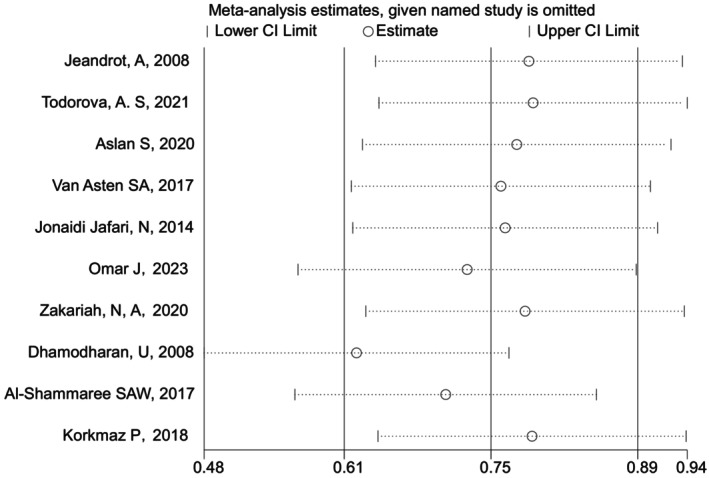
Sensitivity analysis of the meta‐analysis.

We analysed the differences in PCT levels between patients in the IDFU and NIDFU groups. All ten papers included in the study reported the relationship between PCT and IDFU, and there was significant heterogeneity among the included studies (*χ* ^2^ = 54.10, *p* < 0.00001; *I* ^2^ = 83.6%). Consequently, we selected a random effect model SMD with a combined value of 0.79 (95% CI: 0.43–1.14) (Figure 2). This indicated a considerable positive correlation between high PCT and IDFU levels. Egger's test (*t* = 0.41, *p* = 0.691) indicated that there was no publication bias. Analyses of sensitivity indicated that the results are reliable (Figure 3).

**FIGURE 4:**
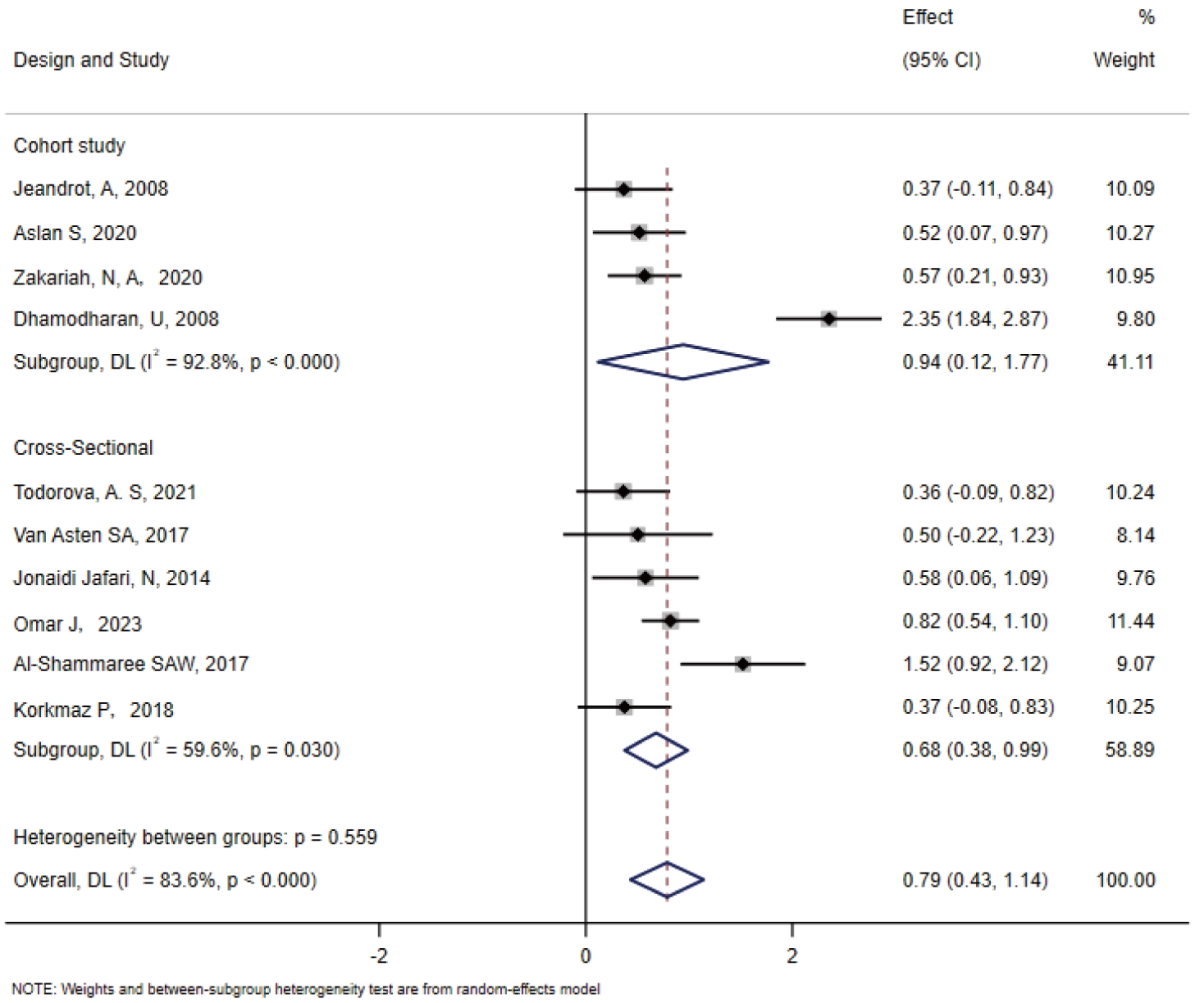
Subgroup analyses of study types.

**FIGURE 5:**
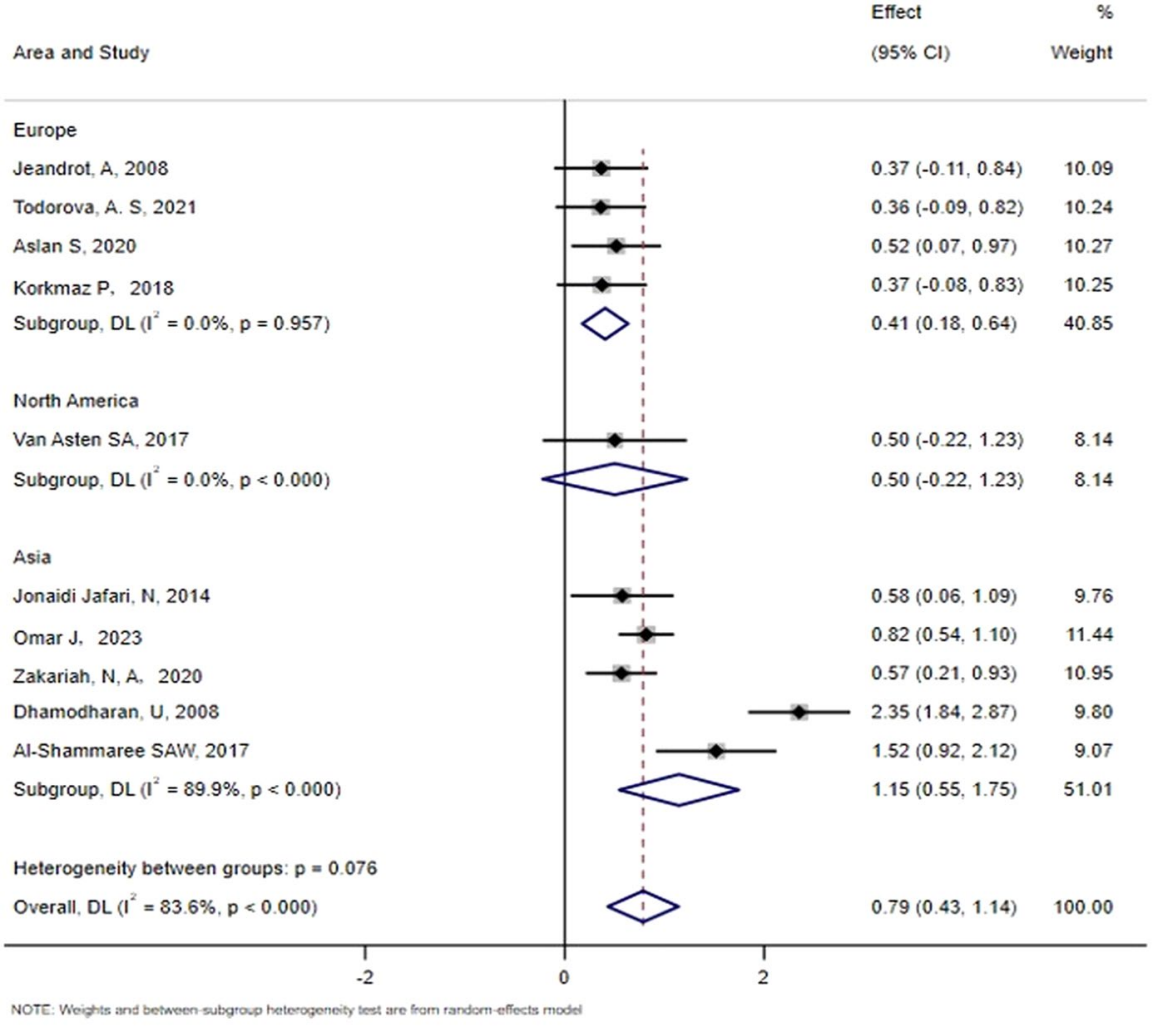
Subgroup analysis of areas.

Separate subgroup analyses were conducted based on the region and study type involved. The subgroup analyses by region revealed that the standardised mean difference (SMD) in Asia was 1.15 (95% confidence interval [CI]: 0.55–1.75), in Europe it was 0.41 (95% CI: 0.18–0.64) and in North America it was 0.50 (95% CI: −0.22‐1.23) (Figure 4). Subgroup analyses of study designs revealed that the standardised mean difference (SMD) for cohort studies was 0.94 (95% confidence interval [CI]: 0.12 to 1.77) and 0.68 (95% CI: 0.38 to 0.98) for cross‐sectional studies (Figure 5).

To identify the source of heterogeneity, random effect model meta‐regression analyses were conducted. The results indicated that BMI (*p* = 0.026) and HbA1c (*p* = 0.016) had a significant effect on the heterogeneity of the relationship between diabetic foot ulcer infection and serum PCT levels; age (*p* = 0.394) and the percentage of males in the sample (*p* = 0.952) were not significant sources of heterogeneity.

## 4 Discussion

In this study, the association between PCT and diabetic foot ulcer infection at this stage was reviewed systematically. In this meta‐analysis, the total SMD of serum PCT in the IDFU group versus the NIDFU group was 0.79 (95% confidence interval [CI]: 0.43–1.14). This meta‐analysis revealed a strong relationship between diabetic foot ulcer infections and PCT.

In our study, PCT levels were substantially higher in the IDFU group compared to the DFU group. PCT is a precursor of calcitonin and is secreted in response to infection by C cells of the thyroid gland as well as neuroendocrine cells of the gut and lungs [21]. PCT has a half‐life of 25–30 h and reaches its peak 6 h after infection onset [22]. In healthy individuals, serum concentrations of procalcitonin are typically low; however, all parenchymal tissues stimulate procalcitonin production, and concentrations rise swiftly in response to bacterial infection. Bacterial endotoxins and lipopolysaccharides directly stimulate PCT production, while inflammatory mediators like TNF‐, IL‐6 and IL‐1 indirectly stimulate PCT production [23, 24]. Park et al. discovered a positive correlation between PCT and the degree of DFI infection, and the level of change in PCT concentration was dependent on the site and degree of infection [8].

The outcomes of regional subgroup analyses suggest that PCT levels in DFU patients may vary across continents, particularly in Asia. It has been reported that Asian populations have relatively poor β‐cell function, resulting in inadequate insulin secretion and diminished glucose‐lowering ability [25]. Differences in economic status and way of life could be one of the causes of these differences [26]. In addition, for individuals with the same BMI, the risk of developing diabetes is 2.5 times higher in Asian populations than in Europe and the United States. This is because body fat in Asian populations tends to accumulate in the internal organs (particularly the liver) and is more likely to induce insulin resistance [27].

At this time, the PCT threshold for distinguishing between infected and non‐infected diabetic foot ulcers remains an important issue [28]. When serum PCT is elevated in patients with diabetic foot, it is also important to consider the possibility of other bone infectious diseases and other comorbidities [29]. Five of the included studies indicated the optimal PCT threshold for the diagnosis of DFU to be 66.55 pg/dL, 0.25 ng/mL, 0.21 ng/mL, 0.11 ng/mL, and 0.5 ng/mL. In the future, it is anticipated that more studies on the optimal biomarkers for determining whether DFU is infectious or not will be conducted so as to provide clinicians with greater assistance in diagnosing DFU.

There are limitations to this meta‐analysis. First, the techniques used to detect PCT values in the included studies were not standardised and were subject to error. Second, articles were restricted to English‐language publications that had been peer‐reviewed; eligible studies reported in other languages and publication categories were possibly excluded. Lastly, there was substantial heterogeneity in sociodemographic and DFU‐related characteristics between and within the study samples, making it difficult to determine the generalisability of the findings.

## 5 Conclusion

As the global economy continues to grow, the number of people with diabetes will increase year after year, and the incidence of IDFU is also increasing year after year. The prolonged course of the disease and the high rate of disability have placed a significant mental and financial burden on the majority of patients. At this stage, accurate diagnosis and differentiation of different types of IDFU remain a significant challenge. This study clarifies the relationship between PCT and IDFU, and future research should investigate the effects of additional variables on PCT levels. Author Contributions: W.W. and P.Z. contributed equally to this work. W.W. led the entire study process, including literature search, screening, data extraction, quality assessment, statistical analysis, and manuscript writing. P.Z. assisted with data extraction, quality evaluation, and manuscript revision. X.N. served as the third reviewer in resolving disagreements and provided clinical guidance. Q.H. conceived and supervised the study, oversaw the research workflow, and critically revised the final manuscript. All authors read and approved the final version of the manuscript. Conflicts of Interest: The authors declare no conflicts of interest.

## Author Contributions

W.W. and P.Z. contributed equally to this work. W.W. led the entire study process, including literature search, screening, data extraction, quality assessment, statistical analysis, and manuscript writing. P.Z. assisted with data extraction, quality evaluation, and manuscript revision. X.N. served as the third reviewer in resolving disagreements and provided clinical guidance. Q.H. conceived and supervised the study, oversaw the research workflow, and critically revised the final manuscript. All authors read and approved the final version of the manuscript.

## Conflicts of Interest

The authors declare no conflicts of interest.

## Data Availability

Data sharing not applicable to this article as no datasets were generated or analysed during the current study.
